# Inhibition of TNFalpha in vivo prevents hyperoxia-mediated activation of caspase 3 in type II cells

**DOI:** 10.1186/1465-9921-6-10

**Published:** 2005-01-21

**Authors:** Florian Guthmann, Heide Wissel, Christian Schachtrup, Angelika Tölle, Mario Rüdiger, Friedrich Spener, Bernd Rüstow

**Affiliations:** 1 Humboldt-Universität zu Berlin, Klinik für Neonatologie, Charité Campus Mitte, D-10098 Berlin, Germany; 2 Westfälische Wilhelms-Universität Münster, Institut für Biochemie, Wilhelm-Klemm-Str. 2, D-48149 Münster, Germany

**Keywords:** Hyperoxia, lung, alveolar type II cells, TNFα, tumour necrosis factor receptor, caspase, apoptosis

## Abstract

**Background:**

The mechanisms during the initial phase of oxygen toxicity leading to pulmonary tissue damage are incompletely known. Increase of tumour necrosis factor alpha (TNFalpha) represents one of the first pulmonary responses to hyperoxia. We hypothesised that, in the initial phase of hyperoxia, TNFalpha activates the caspase cascade in type II pneumocytes (TIIcells).

**Methods:**

Lung sections or freshly isolated TIIcells of control and hyperoxic treated rats (48 hrs) were used for the determination of TNFalpha (ELISA), TNF-receptor 1 (Western blot) and activity of caspases 8, 3, and 9 (colorimetrically). NF-kappaB activation was determined by EMSA, by increase of the p65 subunit in the nuclear fraction, and by immunocytochemistry using a monoclonal anti-NF-kappaB-antibody which selectively stained the activated, nuclear form of NF-kappa B. Apoptotic markers in lung tissue sections (TUNEL) and in TIIcells (cell death detection ELISA, Bax, Bcl-2, mitochondrial membrane potential, and late and early apoptotic cells) were measured using commercially available kits.

**Results:**

In vivo, hyperoxia activated NF-kappaB and increased the expression of TNFalpha, TNF-receptor 1 and the activity of caspase 8 and 3 in freshly isolated TIIcells. Intratracheal application of anti-TNFalpha antibodies prevented the increase of TNFRI and of caspase 3 activity. Under hyperoxia, there was neither a significant change of cytosolic cytochrome C or of caspase 9 activity, nor an increase in apoptosis of TIIcells. Hyperoxia-induced activation of caspase 3 gradually decreased over two days of normoxia without increasing apoptosis. Therefore, activation of caspase 3 is a temporary effect in sublethal hyperoxia and did not mark the "point of no return" in TIIcells.

**Conclusion:**

In the initiation phase of pulmonary oxygen toxicity, an increase of TNFalpha and its receptor TNFR1 leads to the activation of caspase 8 and 3 in TIIcells. Together with the hyperoxic induced increase of Bax and the decrease of the mitochondrial membrane potential, activation of caspase 3 can be seen as sensitisation for apoptosis. Eliminating the TNFalpha effect in vivo by anti-TNFalpha antibodies prevents the pro-apoptotic sensitisation of TIIcells.

## Background

Oxidative stress is an important factor of acute lung injury. Prolonged exposure to high concentrations of oxygen (hyperoxia) during mechanical ventilation represents a life-saving intervention for critically ill patients. However, it also induces oxidative stress to the lung. The development of therapeutic strategies, aiming to prevent lung injury depends on a better understanding of the underlying pathways of hyperoxia-induced pulmonary damage.

Severe, long lasting hyperoxia causes an inflammatory reaction with an influx of inflammatory cells, cell proliferation and hypertrophy, an increase of cytokines, apoptotic activity and subsequent morphologic evidence of lung injury [1]. The first 24 to 48 hrs of oxygen exposure constitute the initiation phase of the pulmonary oxygen toxicity [1]. Even though no morphologic injury has been described during this phase, several changes occur due to the hyperoxic exposure. Highly reactive oxygen species are likely to cause lipid peroxidation, protein and DNA modification that will further cell injury [2,3]. On the other hand, antioxidant enzymes are also induced and may counteract the oxidative stress [4-6]. Perkowski et al. analysed more then 8700 genes during the early response (0 up to 48 hrs) to hyperoxia in total lung of mice. Out of 385 genes in the lung, 175 showed an increased and 210 a decreased expression [6]. These results indicate that the initiation phase of hyperoxic-induced lung injury already marks a very complex process that is still poorly understood. From previous investigations it may be concluded that in response to oxidative stress, the number of endothelial cells strongly decreases in the post-initiation phase, whereas epithelial cells seem to be relative resistant to oxidative stress [1,7]. In contrast, it has also been shown that in response to hyperoxic ventilation [8], emphysema [9], activation of the Fas/FasL system [10], exposure to donors of nitric oxide or hydrogen peroxide [11], hyperoxia and nitric oxide [12], respiratory distress syndrome [13], and hyperoxia-mediated increase of total lung p53 protein expression [14] alveolar type II cells (TIIcells) are severely damaged, culminating in apoptotic death. TIIcells are functionally highly important epithelial lung cells. They are responsible for the metabolism of alveolar surfactant, serve as progenitor cells of type I pneumocytes, and take part in the inflammatory response of the lung [15-17]. Thus, damage and apoptotic elimination of TIIcells will severely alter pulmonary function.

Following the concept that hyperoxic lung injury is a continuous process, we assumed that appropriate metabolic changes of TIIcells start during the initiation phase. This would then, in response to longer lasting severe hyperoxia or an additional stress, merge in apoptosis in the post-initiation phase [18]. Factors which induce such pro-apoptotic sensitisation of TIIcells in the initiation phase are yet unknown.

Elevation of tumour necrosis factor α (TNFα) represents one of the first pulmonary responses to hyperoxia. Pre-treatment of animals with antibodies directed against TNFα reduces hyperoxia-induced lung injury, strongly suggesting a causal relationship between TNFα and hyperoxic lung [19]. TNFα is a classic regulator of cell death by apoptosis or necrosis [20,21]. Cellular response to TNFα is mediated by TNF receptor type I and type II (TNFRI and TNFRII; [22]). Pryhuber et al. [23] studied the contribution of both, TNFRI and TNFRII to hyperoxia-induced lung injury and found that the average length of early survival under hyperoxic conditions is significantly improved in mice that lack the TNFRI (-/-), when compared with wild type or TNFRII (-/-) mice, respectively. However, the blockade of the TNFα receptor function does not protect against pulmonary inflammation and toxicity induced by prolonged hyperoxia [23]. In fact, during prolonged hyperoxia severe lung injury is most likely initiated by additional factors beside TNFα, as the inhibition of TNF receptors does not further affect oxygen-induced mortality [23]. Thus, TNFα and signal transduction via TNFRI seems to be responsible for metabolic changes that regulate the length of survival under short term hyperoxia.

In this paper, we tested the hypothesis that TNFα activates caspases in the initiation phase of pulmonary oxygen toxicity in TIIcells without a significant increase in apoptosis. Since apoptosis is modulated by cell-matrix and cell-cell interactions in cultured TIIcells isolated from animals with acute lung injury [24,25]. we used freshly isolated TIIcells for our study.

## Materials and methods

### Hyperoxia

Wistar rats (body wt 120 g), each in an individual plastic chamber were continuously gassed with 100 % oxygen for 48 hours. Water and food was available *ad libitum*. Preparation of the bronchoalveolar lavage, alveolar macrophages and TIIcells was carried out as previously described [26].

### Immunohistochemistry

Immunohistochemistry and microscopy were carried out as previously described [26]. The following antibodies were used: Rabbit polyclonal anti-rat TNFα antibody from Biosource Europe (Nivelles, Belgium), rabbit polyclonal anti TNFRI antibody raised against a recombinant peptide (amino acids 30–301) including the extracellular domain of TNFRI (Santa Cruz Biotechnology, Heidelberg, Germany), and anti-active caspase 3 polyclonal antibody was from Promega (Mannheim, Germany). Secondary antibodies conjugated with Alexa 499 and Alexa 594 were from Molecular Probes Europe BV (Leiden, Netherlands). Lung tissue sections were labelled with specific antibodies directed against TNFRI, TNFα, caspase 3, and p180 [27], an integral lamellar body-limiting membrane protein (clone 3C9, Covance/Berkeley Antibody, Richmond, CA, USA).

For double staining, the labelled preparations were analysed using a confocal laser scanning microscope (CLSM, Leica Microsystems AG, Wetzlar, Germany), equipped with an argon/krypton laser. Images were taken using a 40 × NA 1.3 oil objective to fluorescent excitation and emission spectra for Alexa 488 (excitation 490 nm, emission 520 nm) and for Alexa 594 (excitation 541 nm, emission 572 nm). With the dual-channel system of the confocal microscope, dual-emission (535/590 nm) images were recorded simultaneously with a scanning speed at 16 s/frame (512 lines). Images were obtained and processed using TCS NT Version 1.5.451 (Leica Microsystems AG, Wetzlar, Germany). As controls, the tissue slides were incubated with the Alexa-labelled second antibodies only. No unspecific binding of the second antibodies occurred (results not shown).

**Figure 6:**
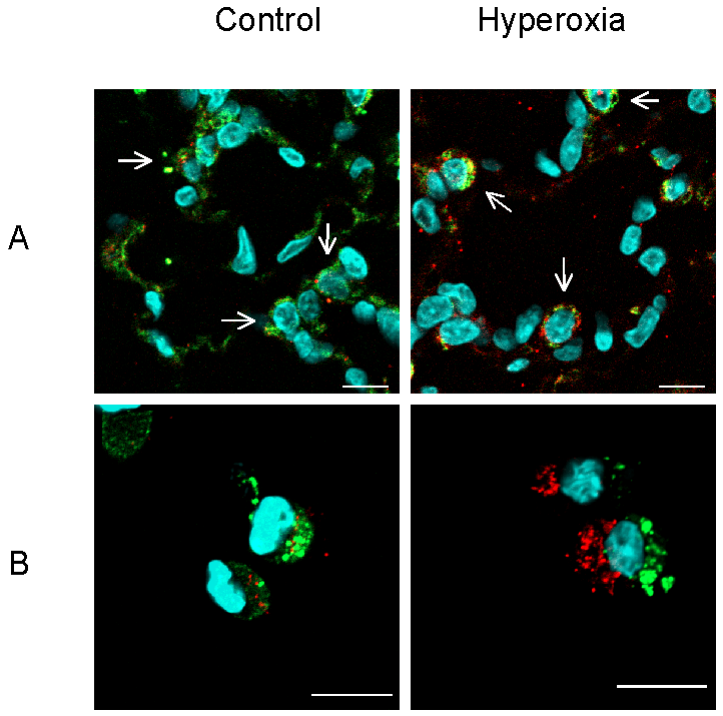
Hyperoxia activates caspase 3 in TIIcells. Rats were kept normoxic (control) or subjected to hyperoxia. Lung sections (A) and freshly isolated TIIcells (B) were immunohistochemically threefold stained as described in *Materials and Methods*. Cell nuclei stained light blue (DAPI). TIIcells are distinguishable by the close proximity of their nuclei to green stained lamellar bodies (A; arrows). Active caspase 3 appears red labelled and is predominantly found in the cytosol of TIIcells upon hyperoxic treatment of rats (A and B). Bar 10 μm;

For threefold staining (Figure 6), fixed lung tissue and freshly isolated TIIcells were incubated in 0.01 M phosphate buffered saline containing 1% (w/v) BSA and 0.3% (w/v) Triton X-100 for 1 hr at room temperature (RT). Detection of lamellar bodies and active Caspase 3 was achieved by incubation with mab 3C9 (20 hrs at 4°C) followed by Alexa 488-labelled goat anti-mouse IgG (2 hrs at RT) and with anti-active caspase 3 followed by Alexa 594-labelled goat anti-rabbit IgG (2 hrs at RT), respectively. Nuclear DNA was stained with 4',6-diamidino-2-phenylindole (DAPI; Molecular Probes Europe BV, Leiden, Netherlands) for 20 minutes at RT.

Laser scanning confocal microscopy was performed using a ZEISS LSM 510 system with Axiovert microscope (Carl Zeiss Jena GmbH, Jena, Germany) with 40×/1.3 Oil Dic or 63×/1.4 Oil Dic objective, equipped with an argon, helium/neon and violet laser set to 488, 543 and 405 nm, respectively. The multitrack standard FITC/Rhodamine/DAPI configuration was selected.

### Determination of apoptosis in lung tissue

#### TUNEL reaction

Sections of rat lung were prepared as described [26]. After deparaffinization and proteinase K-treatment, apoptotic cuts of chromatin DNA were specifically detected by nick end labelling of 3'-OH DNA ends with fluorescein-dUTP using terminal deoxynucleotidyl transferase. (MEBSTAIN Apoptosis Kit Direct, MBL, Naka-ku Nagoya, Japan). Sections were analyzed using a confocal laser scanning microscope as described above for double staining.

### Determination of apoptosis in freshly isolated TIIcells

#### Cell Death Detection ELISA

Cytoplasmic histone-DNA fragments were quantified using the Cell Death Detection ELISA (Roche, Mannheim, Germany).

#### Flow cytometry

We used the TACS™ Annexin V-FITC Detection kit (R&D Systems, Wiesbaden, Germany) to quantify the population of early and late apoptotic cells in percent of total cells. The tests were performed according to the protocols of the manufactures.

### Determination of caspase activities

Activities of caspases 3, 8 and 9 were determined in the lysates of 8 × 10^6 ^TIIcells for each group and for each caspase with the Colorimetric assays from R&D Systems Inc. (Wiesbaden, Germany). When the pro-caspases had to be determined, an aliquot of the lysate (corresponding to 2 × 106 cells) was preincubated with 0.1 μg granzyme (Calbiochem, Bad Soden, Germany; dissolved in 5 μl 0.9% NaCl) for 30 min at 37°C.

### Determination of NF-κB activation

#### Immunocytochemistry

Activation of NF-κB was measured by immunocytochemistry using a monoclonal anti-NF-κB-antibody (MAB3026, Chemicon International, Temecula, USA), that recognises an epitope which includes the nuclear location signal of p65, the DNA binding subunit mainly responsible for the strong gene-inductory potential of NF-κB. Thus, only the activated form of NF-κB was measured. The semiquantitative estimation of NF-κB subunit by confocal microscopy was carried out as recently described in detail [28].

#### Immunoblotting

Translocation of NF-κB to the nucleus was assessed as described by Li et al. by immunoblotting of nuclear extracts using a rabbit polyclonal antibody (biomol GmbH, Hamburg, Germany) directed against the p65-subunit [29].

#### Electrophoretic mobility shift assay

(EMSA) was employed to detect the activated transcription factor NF-κB. Because this method is based on the binding of the transcription factors to their specific DNA recognition sequences, it is highly specific. Labelling of the NF-κB consensus oligonucleotide and handling of the assay were as described by the manufacturer (Gel Shift Assay Systems, Promega GmbH, Mannheim, Germany). Briefly, 50 micrograms of TIIcell nuclear extract were preincubated in reaction buffer for 10 minutes at RT. A [32P]-labelled oligonucleotide (Promega GmbH, Mannheim, Germany) which contains DNA binding sites for NF-κB transcription factors was then added to the reaction mixture and incubated for 20 minutes at RT. The complexes were separated on a 4% polyacrylamide gel that was dried and exposed to autoradiography. The specificity of the DNA-binding protein for the putative binding site was established by competition experiments using unlabelled NF-κB consensus oligonucleotide.

### Intratracheal application of anti-TNFα antibodies

Wistar rats were lightly anaesthetised by inhalation of ether. The rats obtained intratracheally 50 μg goat IgG (PERBIO SCIENCE, Bonn, Germany) or 50 μg goat polyclonal anti-rat TNFα antibody (Santa Cruz Biotechnology, Heidelberg, Germany) per animal, respectively. Thereafter the rats were kept at hyperoxic conditions as described in hyperoxia. After 48 hrs, the TIIcells were isolated and TNFRI expression and caspase-3 activity were determined as described above.

### Other methods

For the determination of the TNFα concentration in TIIcells, macrophages, plasma or cell-free bronchoalveolar lavage, we used a commercially available ELISA kit from Biosource (Ratingen, Germany). The determination of different apoptotic markers [18], Western blot analysis [17], mRNA isolation and Real-time quantitative PCR reaction for the determination of the expression of mRNAs in TIIcells was described in detail previously [26]. GSH, GSSG and GSH-reductase were determined by HPLC with subsequent fluorescence detection as previously described [17].

### Statistical analysis

Differences between two groups were assessed using the Student's t-test. Probability values < 0.05 (two-tailed) were considered significant (see legends of Tables and Figures).

## Results

### Hyperoxia-induced changes in lung tissue

**Figure 1:**
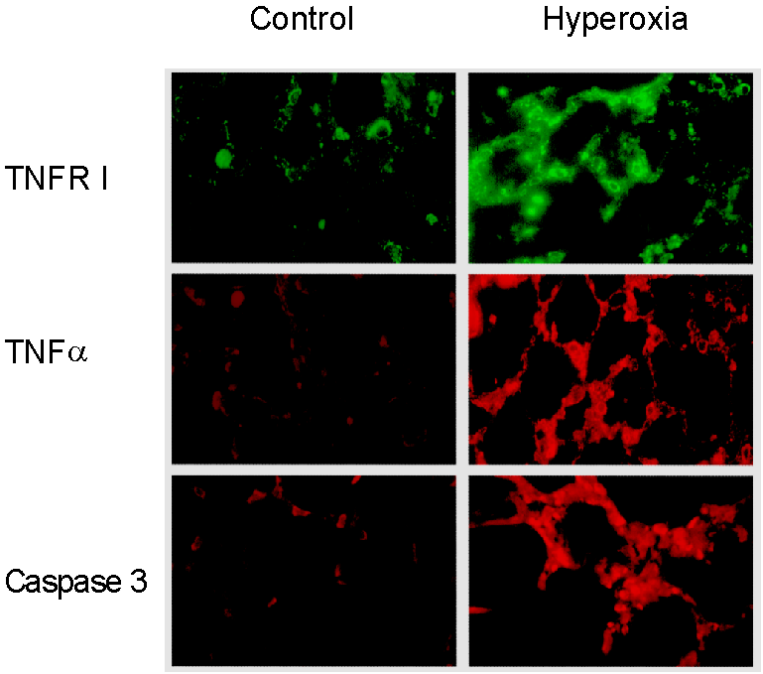
Hyperoxia increases the expression of TNFRI, TNFα and caspase 3 in vivo. Lung sections of normoxic (control) and hyperoxic rats were prepared as described in *Materials and Methods *and were labelled by single immunofluorescence with antibodies directed against TNFRI, TNFα or active caspase 3

**Figure 2:**
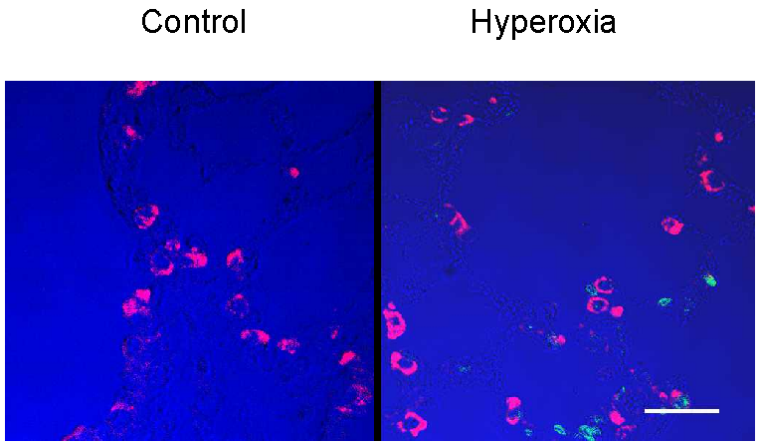
No significant apoptosis was found in TIIcells upon hyperoxia in vivo for 48 hrs. Lung tissue of normoxic rats (left) and of hyperoxic rats (right) were tested for DNA fragmentation by TUNEL reaction as described in *Materials and Methods*. The positive TUNEL reaction is represented by green fluorescence. The presence of lamellar bodies is indicated by red fluorescence. Pseudo-colour blue was used to highlight the contours of lung tissue. Bar: 25 μm.

To test the general usefulness of our hypothesis, we first characterised the oxygen-induced changes in lung-tissue-sections. Immunohistochemically, we observed a clear increase in TNFα, TNFRI, and caspase 3 activities in lung tissue of hyperoxic rats in relation to normoxic animals in the initiation phase (Figure 1). Albeit the increase of these pro-apoptotic parameters in lung tissue corroborated our hypothesis, this approach does not allow to identify the participating cell types. Furthermore, we tested lung sections for DNA-degradation products using the TUNEL reaction (Figure 2). Here, TIIcells are distinguishable from other cells of the lung by their content of lamellar bodies. Lamellar bodies were immunohistochemically labelled in lung sections with an antibody directed against the 180-kDa lamellar body-limiting membrane protein (red, Figure 2). Upon hyperoxia, we found sporadically a fragmentation of DNA (green, Figure 2), but no co-localisation of the 180-kDa lamellar body-limiting membrane protein and DNA-fragments. The intensity of red lamellar body stain increased in lung sections from hyperoxic rats. This is in good accordance with previously published electron micrographs showing swollen and deformed lamellar bodies after hyperoxia [30]. From these results, we conclude in agreement with the literature [1,7,25]. that sublethal hyperoxia of rats did not induce apoptosis in TIIcells *in vivo*; at least not in the initiation phase.

The results indicate that apoptotic parameters as are TNFα content, TNFRI expression and caspase-3 activity increase in lung tissue during the initiation phase but do not induce TIIcell apoptosis *in vivo*. Following our hypothesis, we examined whether TIIcells undergo oxidative stress at our conditions, and whether an increase of TNFα, TNFRI expression, and caspase-3 activity appears in isolated TIIcells.

### Hyperoxia-induced oxidative stress of freshly isolated TIIcells

The determination of cellular GSH, oxidised GSH (GSSG), and the activity of the GSH-reductase showed the oxidative burdening of TIIcells. In response to hyperoxia, the GSSG content significantly increased and the GSH reductase activity significantly decreased (Table 1). The GSH content in freshly isolated TIIcells has rarely been determined. In our TIIcell population, the GSH content differs in relation to previously published data of freshly isolated TIIcells from rat [31] and rabbit [32] by the factor of about 2 and 4, respectively. These differences might be explained by different methods of TIIcell isolation and by species specificity.

**Table 1:** Effect of hyperoxia on GSH reductase activity, and on GSH and GSSG content in TIIcells

	Control	Hyperoxia
GSH reductase (% of control)	100	76 ± 10*
GSH (μmol/mg protein)	0.022 ± 0.015	0.020 ± 0.008
GSSG (μmol/mg protein)	0.005 ± 0.0015	0.013 ± 0.001*
GSH/(GSH+GSSG) (ratio)	0.81	0.61

Activity of glutathione (GSH) reductase and concentration of GSH and GSSG were determined in freshly prepared TIIcells of rats exposed to air (control) or oxygen for 48 hrs (hyperoxia) as described in *Materials and Methods*. Values are means ± standard deviation of n = 3 independent experiments. Asterisk indicates a significant difference to control (*p *< 0.05).

The ratio GSH/(GSH+GSSG) is one of the most sensitive parameters to describe oxidative burdening. Our values are comparable to the values found by van Klaveren et al. in freshly isolated type II cells (0.816 versus 0.815) [30]. This ratio decreased in response to hyperoxia in vitro to 0.74 [31] and in our in vivo approach to 0.61 (Table 1). With respect to the published data, we conclude that our treatment induced oxidative burdening in freshly isolated type II cells.

### Hyperoxia activates NF-κB

**Figure 3:**
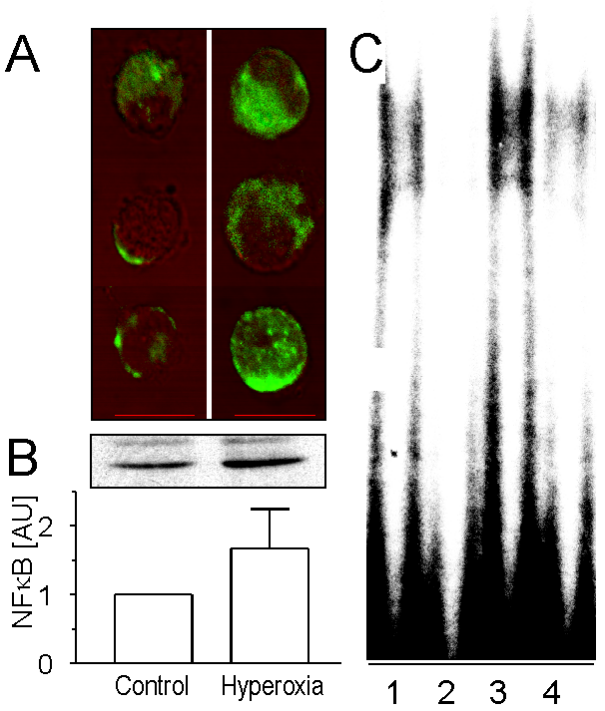
Activation of NF-κB in TIIcells and its enrichment in the nuclear protein fraction after hyperoxic treatment of rats. Freshly isolated TIIcells from normoxic (control) and hyperoxic rats were prepared for immunocytochemistry, SDS-PAGE and immunoblotting as described in *Materials and Methods*. A, activation of NF-κB was measured by immunocytochemistry using a monoclonal anti-NF-κB-antibody overlapping the nuclear localisation signal of the p65 subunit in the NF-κB heterodimer. Activated NF-κB in the nucleus and cytosol was quantified as described recently in detail [28]. The signal of activated NF-κB in the nucleus increased 4.7 fold in respose to hyperoxia (n = 12; p < 0.05). Bar: 10 μm. B, the nuclear protein fraction [52] of TIIcells was subjected to SDS-PAGE and immunoblotting. The p65 subunit of NF-κB was visualised using a rabbit polyclonal antibody, and its expression was densitometrically estimated. Values of n = 4 independent experiments are given as mean ± SD in arbitrary units (control = 1). C, Electrophoretic mobility shift assay for NF-κB in freshly isolated TIIcells from normoxic (lanes 1, 2) and hyperoxic (lanes 3, 4) rats. Signal competition upon addition of unlabelled oligonucleotide (lanes 2, 4).

NF-κB activation has been described as an indicator of oxidative stress [33,34]. In response to sublethal hyperoxia, the content of activated NF-κB in TIIcells clearly increased (Figure 3A). The picture shows that the larger portion of activated NF-κB seems to be localised in cytosol. The semiquantitative determination of activated NF-κB showed a significant increase (control: 12.7 ± 9.0; hyperoxia: 59.5 ± 11.6; n = 12; p < 0.01) in the nucleus of TIIcells in response to hyperoxia. In the cytosol, there was also an increase of activated NF-κB (1.6-fold), however, this difference curtly fails the level of significance. Additionally, we detected a translocation of the NF-κB-subunit p65 into the nuclear protein fraction as estimated by Western blot analysis (Figure 3B). Hyperoxia increased the content of the p65-subunit in the nuclear protein fraction of TIIcells 1.68-fold (SD 0.58, n = 4) compared to normoxic control, but the difference did not reach significance. Whether the immunoreactive band above the p65-subunit in the Western blot of the hyperoxic group is non-specific or a possible post-translational modification can not be decided. In Figure 3C we confirm the hyperoxia induced activation of NF-κB by EMSA of the nuclear protein fractions of TIIcells freshly isolated from control (lanes 1, 2) and hyperoxic (lanes 3, 4) rats. Addition of unlabelled specific oligonucleotide clearly competes with the [32P]-labelled probe confirming the specificity of the bands.

### Hyperoxia increases synthesis and secretion of TNFα by TIIcells

**Figure 4:**
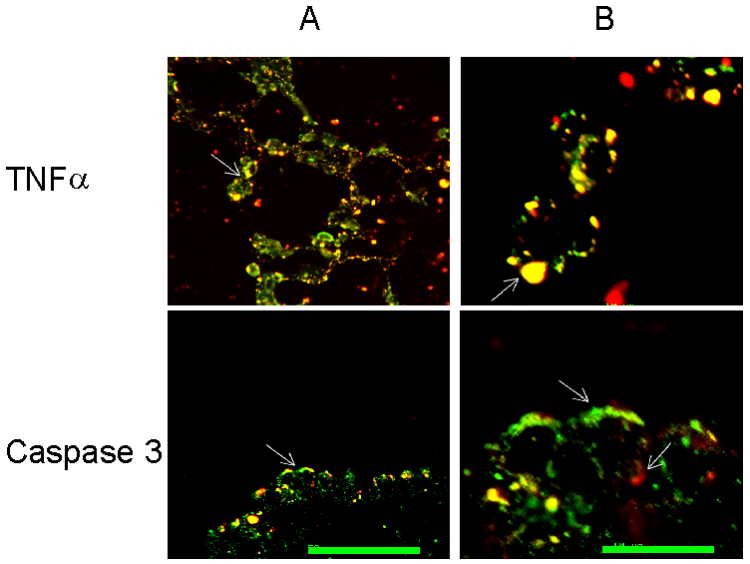
TNFα is localised in lamellar bodies and caspase 3 in the cytosol of TIIcells. After hyperoxia, rat lungs were fixed and the sections were immunohistochemically double labelled as described in *Materials and Methods*. A: bar 50 μm; B: higher magnification of the indicated area of A (arrow); bar 10 μm. By confocal microscopy, TIIcells were identified by the green labelling of lamellar bodies (see Methods). The red labelled TNFα appears yellow (arrow in B) when co-localised in lamellar bodies as shown in two TIIcells in B.

The concentration of TNFα significantly increased in response to hyperoxia of rats in plasma, alveolar fluid, lung macrophages and TIIcells (Table 2) as determined by ELISA. Flow cytometric analysis of the TNFα content in freshly isolated TIIcell preparations from control and hyperoxic rats confirmed the increase of the TNFα concentration in macrophages and TIIcells. In response to hyperoxia, TNFα increased in TIIcells 1.45-fold and in macrophages 1.87-fold compared to control. In TIIcells, TNFα seems to be localised in lamellar bodies (Figure 4), whereas cytoplasmic caspase 3 showed no co-localisation with lamellar bodies as expected. The latter result attaches value to the histochemically detected localisation of TNFα in lamellar bodies, because it is unlikely an artefact. The spontaneous secretion of TNFα by TIIcells significantly increased in response to hyperoxia (Table 2).

**Table 2:** Effect of hyperoxia on the TNFα content in different specimen from rat

	Control	Hyperoxia
Plasma (pg/ml)	106 ± 31	149 ± 11
Macrophages (ng/mg protein)	11.4 ± 3.7	19.4 ± 2.8*
TIIcells (ng/mg protein; n = 6)	18.5 ± 2.1	27.2 ± 6.7*
Bronchoalveolar lavage (pg/ml)	109 ± 1	172 ± 38*
Spontaneous secretion of TNFα by TIIcells (ng × mg cell protein^-1 ^× hr^-1^)	6.3 ± 1.3	21.2 ± 7.5*

Concentration of TNFα was determined in plasma, macrophages, TIIcells and bronchoalveolar lavage of rats exposed to air (control) or oxygen for 48 hrs (hyperoxia) by ELISA as described in *Materials and Methods*. Spontaneous secretion of TNFα was measured in cell-free supernatant upon incubation of freshly isolated TIIcells in DMEM for 30 min at 37°C. TNFα concentrations are given as means with standard deviation of n = 3 independent experiments unless stated otherwise. Asterisk indicates a significant difference to control (*p *< 0.05).

### Hyperoxia induces the expression of TNFRI and activates caspases in TIIcells

**Figure 5:**
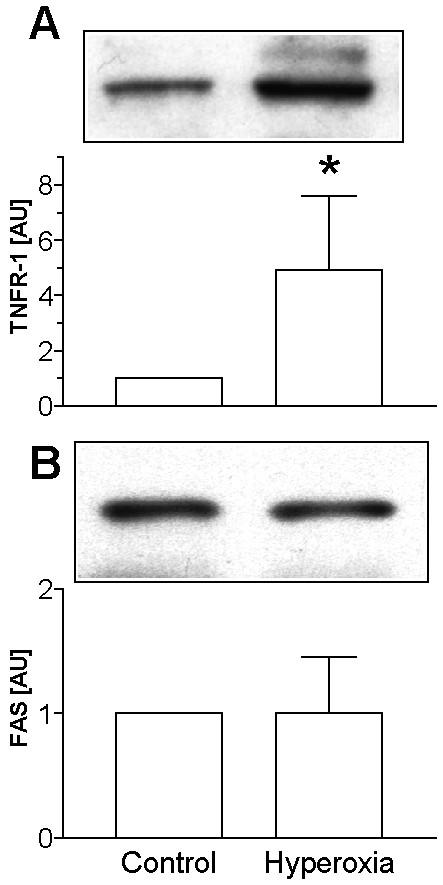
Hyperoxia increases the expression of TNFRI but not of Fas in TIIcells. For the expression analysis of TNFRI and Fas the membrane fraction of freshly isolated TIIcells was prepared. TNFRI (A) and Fas (B) were visualised by Western blot technique, and their expression was densitometrically determined. Values of n = 6 independent experiments are given as means ± SD in arbitrary units (control = 1). Asterisk indicates a significant difference to control (*p *< 0.05).

Hyperoxia induced an significant increase of TNFRI on TIIcells (4.9-fold ± 2.7, n = 6), whereas the expression of Fas, a member of the same receptor family, did not change (Figure 5). TNFRI-mediated action of TNFα depends on the so called "death domain" representing a part of the intracellular segment of the receptor protein responsible for the activation of pro-caspase 8. Caspase 8 in turn can activate pro-caspase 3, a feature previously reviewed [35-37]. We show *in situ *that the activation of caspase 3 actually occurs in TIIcells and that caspase activation as a response to an unspecific stress, e.g. isolation, can be excluded (Figure 6). This staining technique excepts cytoplasm and membranes, thus, cells can hardly be delimited. However, TIIcells are identifiable by the immediate proximity of their nuclei to lamellar bodies (green).

As shown in Table 3, in TIIcells the activity of caspase 8 and 3 increased in response to hyperoxia, whereas the activity of caspase 9 did not change. Pre-incubation with granzyme activated pro-caspases and resulted in an increase of caspase 8 and -3 activities in control and hyperoxic TIIcells (Table 4). However, the activation in control cells clearly exceed that in hyperoxic TIIcells, indicating that pro-caspases were already, at least in part, activated in response to hyperoxia.

**Table 3:** Effect of hyperoxia on the activity of caspases in TIIcells

	Control	Hyperoxia
Caspase 8 (n = 6)	100	143 ± 14*
Caspase 3 (n = 6)	100	168 ± 23*
Caspase 9 (n = 3)	100	108 ± 11

Caspase activity in freshly prepared TIIcells of rats exposed to air (control) or oxygen for 48 hrs (hyperoxia) was determined colorimetrically as described in *Materials and Methods*. Values are means ± S.D. given in arbitrary units (control = 100). Asterisk indicates a significant difference to control (*p *< 0.05).

**Table 4:** Effect of granzyme-treatment on the activity of caspases in TIIcells from normoxic and hyperoxic rats

	Control		Hyperoxia	
		
Granzyme	**-**	**+**	**-**	**+**
Caspase 8	100	155 ± 7*	100	110 ± 4*
Caspase 3	100	216 ± 14*	100	155 ± 10*

Caspase activity in freshly prepared TIIcells of rats exposed to air (control) or oxygen for 48 hrs (hyperoxia) was determined colorimetrically as described in *Materials and Methods*. In some experiments, cell lysates were incubated in the presence of granzyme prior to determination of caspase activity (see *Methods*). Values are means ± s. d. given in arbitrary units (values without granzyme = 100) of n = 3 independent experiments. Asterisk indicates a significant difference between granzyme-treated and untreated samples (*p *< 0.05).

### Anti-TNFα in vivo prevents hyperoxia-driven increase in TNFRI and in active caspase 3

In order to demonstrate the causality between hyperoxia and TNFRI-mediated activation of caspases, we attempted to bind TNFα by anti-TNFα antibodies. Table 5 shows that a single intratracheal application of anti-TNFα antibodies immediately preceding hyperoxic treatment prevents the hyperoxic-induced increase of TNFRI expression and caspase-3 activity in freshly isolated TIIcells. These results indicate that both TNFRI expression and caspase-3 activation were induced by TNFα. This corroborates the concept that in a cascade starting by the TNFα/TNFRI-interaction caspase 8 is activated which in turn activates caspase 3.

**Table 5:** Effect of intratracheal application of anti-TNFα antibodies on hyperoxic induced parameters of TIIcells

	Hyperoxia	
	without	with anti-TNFα antibody
TNFRI	100	26 ± 19*
Caspase 3 activity	100	64 ± 5*

Rats obtained intratracheal 50 μg goat-IgG (without) or 50 μg goat polyclonal anti-TNFα antibodies (with anti-TNFα antibody) preceding hyperoxic treatment. Hyperoxia of rats was carried out as described in *Material and Methods*. Values are means ± SD of n = 3 independent experiments given in percent (hyperoxia without anti-TNFα antibody = 100). Determination of TNFRI (Western blot) and of caspase 3 activity (colorimetrically) was as described in *Material and Methods*.). Asterisk indicates a significant difference compared to control (*p *< 0.05).

### Hyperoxia upregulates genes of TNFRI, TNFα and caspases 3 and 8

The content of individual mRNAs in TIIcells was determined by Real-time PCR. In agreement with microarray analysis in total lung of mice [6] we found that the amount of GAPDH mRNA did not change in the first 48 hrs of hyperoxia (results not shown). Therefore, GAPDH was used as a house keeping gene. In contrast, Ho et al. found a small but significant increase of GAPDH mRNA in lung tissue [5]. This difference may be caused by different base material (TIIcells versus lung tissue) and methodical differences (Taqman versus densitometry of autoradiographs).

In response to sublethal hyperoxia the mRNA content of TNFα, TNFRI and caspases increased (Table 6). The increment of caspase-8 mRNA was not significant, but even a ΔΔc_t _of -1.15 indicates a 2.2-fold increase of mRNA content.

**Table 6:** Effect of hyperoxia on the mRNA content of TNFα, TNFRI, and caspases in TIIcells

	number of cycles (Δc_t_)		increase of mRNA	
		
	Control	Hyperoxia	ΔΔc_t_	-fold
TNFα	3.66 ± 0.66	0.93 ± 0.30*	-2.73	6.6
TNFRI	5.02 ± 0.19	4.44 ± 0.11*	-0.58	1.5
Caspase 8	10.3 ± 0.89	9.15 ± 0.35	-1.15	2.2
Caspase 3	5.33 ± 0.25	4.29 ± 0.48*	-1.04	2.1

The mRNA of three animals per group was isolated and combined. The content of specific mRNA was determined using Real time-PCR. In parallel, GAPDH was determined in each run as internal standard. The values are given as number of GAPDH-corrected cycle (Δc_t_) ± SD of n = 3 determinations. ΔΔc_t _is the difference of the number of cycles between control and hyperoxia; a decrease of Δc_t _indicates an increase of the mRNA, also given as a factor of increase. Asterisk indicates a significant difference to control (*p *< 0.05).

### Hyperoxia of rats does not induce apoptosis in freshly isolated TIIcells

As mentioned above in this section, no increase of apoptosis was detected in TIIcells in response to sublethal hyperoxia by immunohistochemistry (Figure 2). To confirm this result, we analyzed biochemical parameters of apoptosis in freshly isolated TIIcells. In agreement with our immunohistochemical results, sublethal hyperoxia of rats did not increase apoptosis in freshly isolated TIIcells (Table 7). Hyperoxia was without effect on anti-apoptotic Bcl-2, and cytosolic cytochrome C, although the pro-apoptotic Bax increased and the mitochondrial transmembrane potential slightly decreased (Table 7). In accordance with these results, the activity of caspase 9 did not change. Therefore, activation of caspase 3 seems to be catalysed by caspase 8.

**Table 7:** Effect of hyperoxia on apoptotic parameters in TIIcells

	Control	Hyperoxia
Bcl-2 (n = 5)	100	96 ± 8
Bax (n = 5)	100	134 ± 28*
Cytochrome c (n = 5)	100	102 ± 15
Mitochondrial membrane potential	100	81 ± 8*
Early apoptotic TIIcells	100	101 ± 26
Late apoptotic TIIcells	100	94 ± 22
Cell death detection ELISA	100	104 ± 13

Several apoptotic parameters were determined in freshly prepared TIIcells of rats exposed to air (control) or oxygen for 48 hrs (hyperoxia) as described in *Materials and Methods*. Values are means ± S.D. given in arbitrary units (control = 100) of n = 3 independent experiments unless stated otherwise. Asterisk indicates a significant difference to control (*p *< 0.05).

### Hyperoxia of rats followed by normoxia reduced caspase 3 activity but did not increase apoptosis in TIIcells

After hyperoxia for 48 hrs, we detected an activation of caspase 8 and caspase 3. Much to our surprise, we found no increase in apoptosis of TIIcells although the activity of these caspases increased. However, apoptosis might occur later than 48 hrs and will not necessarily arise concomitantly with caspase activation. Thus, following hyperoxia the animals were kept for 24 and 48 hrs under normoxic conditions to test TIIcells for appearance of apoptosis at a later time point.

During normoxia, caspase 3 activity gradually decreases compared to control, whereas apoptosis did not change significantly as detected by cell death detection ELISA and by the number of early and late apoptotic cells (Table 8).

**Table 8:** Apoptotic parameters upon sublethal hyperoxia of rats followed by normoxia

	Hyperoxia followed by normoxia for		
	48 hrs hyperoxia	24 hrs normoxia	48 hrs normoxia
Caspase 3 activity	171 ± 18*	97 ± 16	73 ± 29
Early apoptotic cells	102 ± 13	95 ± 14	109 ± 26
Late apoptotic cells	106 ± 29	125 ± 25	85 ± 17
Cell death detection ELISA	97 ± 23	104 ± 6	95 ± 14

Activity of caspase 3 and parameters of apoptosis detected in freshly prepared TIIcells of rats exposed to oxygen for 48 hrs (hyperoxia) followed by normoxia for 24 or 48 hrs as described in Materials and Methods. Values are means ± S.D. given in arbitrary units (normoxic control = 100) of n = 3 independent experiments. Asterisk indicates a significant difference compared to control (*p *< 0.05)

## Discussion

Short-time hyperoxia, as used in this experiments represents the initiation phase of lung injury [1]. We started our investigations with the aim to characterise metabolic changes in TIIcells taking place in this phase. On the one hand, these changes should be less complex than in post-initiation phases. On the other hand, therapeutic interventions to avoid or minimise hyperoxia-induced lung injury should focus in particular on this phase, because morphologic injury of lung tissue, inflammation, and death of lung cells originate from here. Furthermore, the metabolic changes in the initiation phase may, at least in part, be still reversible.

For the first time, we show that sublethal hyperoxia causes not only an increase in TNFα concentration in lung tissue (as previously published [38,39]), but also in freshly isolated TIIcells and that this increase is combined with an enhanced expression of TNFRI and an activation of caspase 3.

It has been widely assumed that macrophages are the source of TNFα in alveolar fluid [40]. Our findings provide evidence that hyperoxic treatment of rats provokes a rise in cellular TNFα not only in alveolar macrophages [41]. We show that the TNFα-gene is up-regulated in TIIcells; in parallel, the TNFα-protein content increased. To the best of our knowledge, our data suggests for the first time that TNFα is localised in lamellar bodies. In the light of this assumption, the secretion of TNFα as a constituent of the lamellar bodies by TIIcells might represent a significant contribution to the increased TNFα-content in alveolar fluid. Whether the small increase of the TNFα concentration in plasma observed by us indicates a beginning systemic inflammation or rather reflects a transfer of TNFα from the alveolar space to plasma, remains open (Table 2).

Cellular effects of TNFα are mediated mainly by its specific receptor, TNFRI. In agreement with our hypothesis, the expression of TNFRI in TIIcells is up-regulated on mRNA and protein level in response to sublethal hyperoxia, while the Fas-expression did not change, although both receptors belong to the same family (Figure 5). It may be speculated that a parallel increase of effector and specific receptor always occurs when the cellular metabolism in TIIcells can be affected by an autocrine mechanism. The increase of Fas/Fas-Ligand might be characteristic in post-initiation phases.

The intracellular part of the transmembrane TNFRI protein contains the "death domain" which is responsible for the activation of caspase 8. This can trigger the path to the final part of apoptosis via activation of caspase 3 [36]. In line with this, we found enhanced levels of caspases 8 and 3, yet caspase 9 was not activated. The latter result is corroborated by the absence of an increased mitochondrial cytochrome C release. The data presented here demonstrate that sublethal hyperoxia of rats did not induce apoptosis in TIIcells despite of the increase in TNFα content, TNFRI expression, and activation of caspase 3.

To check whether this pro-apoptotic state in TIIcells is indeed triggered by TNFα and whether it may be reversible, anti-TNFα antibodies were administered intratracheally prior to hyperoxic exposure. We show that this treatment completely prevented hyperoxic-induced increase of TNFRI expression and caspase 3 activation.

It is a widely accepted concept that activation of caspase 3 marks the "point of no return" in the pathway of apoptotic death of mammalian cells. However, we found that neither in lung tissue nor in freshly isolated TIIcells apoptosis occurs in response to sublethal hyperoxia despite the significant activation of caspases 8 and 3. In other words, the increase of caspase 8 and 3 in response to sublethal hyperoxia did not mark the "point of no return" in TIIcells. This interpretation of our results is strongly corroborated by Perfettini and Kroemer [37]. They summarised that caspase inhibition does not avoid but actually encourage death in TNF induced shock, indicating that caspase activation is not basically synonymous with apoptotic cell death. It could be argued that no apoptosis was found in freshly isolated TIIcells because the increase of caspase 3 activity and the increase of apoptotic parameters does not occur concomitantly. However, we found no increase in apoptosis of TIIcells in a normoxic period of 24 and 48 hrs directly succeeding the hyperoxic treatment of animals, whereas the caspase 3 activity gradually decreased. These results indicate that caspase 3 activation in response to sublethal hyperoxia is reversible and does not kill TIIcells essentially.

The reason why the activation of caspases did not induce apoptosis of TIIcells is not clear. On the one hand, activation of NF-κB has often been implicated as an anti-apoptotic event [42-45], and TNFα induces also the expression of anti-apoptotic TNFα-receptor-associated-factors in lung cells and might inhibit by this way TNFα-induced cell death or apoptosis [46]. Therefore, it may be assumed that both NF-κB activation and expression of anti-apoptotic TNFα-receptor-associated-factors arrest the hyperoxia-induced metabolic changes of TIIcells in the pro-apoptotic state. On the other hand, it can not be excluded that the extend of caspase 3 activation (1.68-fold compared to control) is not high enough to induce apoptosis of TIIcells although a 1.8-fold increase of caspase 3 is combined with apoptosis in total lung of a hyperoxia/pneumonia model [47]. We hypothesise that caspase 3 activation is then followed by apoptosis, when its activation occurs *via *strong mitochondrial damage resulting in cytochrome c release and caspase 9 activation in post-initiation phases of pulmonary oxygen toxicity. This concept is supported by recent findings that mitochondrial cytochrome c release is a key event in hyperoxia-induced lung injury [48]. In fact, the mitochondrial membrane potential and Bax significantly changed in TIIcells in the initiation phase of hyperoxic lung injury, but without cytochrome c release or activation of caspase 9.

Recently, it has been shown that in response to severe hyperoxia of mice apoptosis and necrosis contribute to an extensive cell death; p53, bax, bcl-x, and Fas increased in mRNA and protein level, but the activity of caspase 3 and caspase 1 did not change [49]. This independence of apoptosis from caspase activities in the lung has also been shown in freshly isolated TIIcells. Previously, we showed that an increase of TIIcell-apoptosis in response to vitamin E deficiency of rats is independent of caspase activation [18]. Furthermore, using p53-deficient and Fas-null mice, Barazzone et al. [49] showed that Fas and p53 activation exhibits no linkage to lung injury in response to severe hyperoxia of mice. Contrariwise, it has also been shown that Fas activation in vitro [50] and in vivo results in TIIcell apoptosis and lung inflammation [10,51]. These results confirm that hyperoxic-induced lung injury is multifactorial, and that the elimination of one factor in the network of factors activated in the post-initiation phases often can not avoid lung injury in response to severe hyperoxia.

In summary, TIIcells were not killed in the initiation phase of pulmonary oxygen toxicity. More precisely, its pro-apoptotic sensitisation is the background that, together with an additional stress factor, hyperoxia causes lung injury probably by apoptotic elimination of TIIcells. In agreement with this idea, we showed that the combination of the stress factors hyperoxia and vitamin E deficiency increases TIIcell apoptosis [18]. Taking into account that premature neonates exhibit vitamin E deficiency, it is consequential that ventilation of premature neonates suffering from respiratory distress syndrome with high levels of inspired oxygen amplifies lung injury, which is associated with TIIcell apoptosis [13].

## Conclusions

In the initiation phase of pulmonary oxygen toxicity, an increase of TNFalpha and its receptor TNFR1 leads to the activation of caspase 8 and 3 in TIIcells. Together with the hyperoxic induced increase of Bax and the decrease of the mitochondrial membrane potential, activation of caspase 3 can be seen as sensitisation for apoptosis. Eliminating the TNFα effect in vivo by anti-TNFα antibodies prevents the pro-apoptotic sensitisation of TIIcells.

## List of abbreviations

DAPI – 4',6-diamidino-2-phenylindole; EMSA – electrophoretic mobility shift assay; GSH – gluthatione; GSSG – oxidised gluthatione; NF-κB – nuclear factor-κB; TIIcell – type II pneumocyte; TNFα – tumour necrosis factor α; TNFR – TNFα receptor; TUNEL – terminal transferase dUTP nick end labelling

## Authors' contributions

HW carried out the immunohistochemistry, CS performed the mRNA analysis, AT carried out the protein measurements and participated in data analysis, MR applicated anti TNFα antibodies intratracheally, FS participated in the mRNA analysis and the design of the study, FG and BR conceived of the study, participated in its design and co-ordination, analysed the data and wrote the manuscript. All authors read and approved the final manuscript.
